# Unearthing forensic genetics: preliminary results on the ability to generate DNA profiles from buried human biological samples

**DOI:** 10.1007/s00414-026-03775-4

**Published:** 2026-03-25

**Authors:** Susana Gracia-de-Lucas, César López-Matayoshi, Manuel Lozano-García, Cláudia Gomes

**Affiliations:** 1 Faculty of Law, Complutense University of Madrid (UCM), 28040 Madrid, Spain; 2 Legal Medicine, Psychiatry and Pathology Department, Medicine School, Complutense University of Madrid (UCM), 28040 Madrid, Spain; 3 Department of Cellular and Molecular Sciences, Faculty of Sciences, Universidad Peruana Cayetano Heredia, Lima, 15102 Peru

**Keywords:** Degradation, Inhibition, Allelic loss, Saliva, Blood, Soil

## Abstract

Burying objects with biological material is a common procedure in many crimes, intending to hide and/or accelerate the process of degradation of the possible biological information they may contain. In this sense, the central question of this preliminary study concerns investigating the impact of a burial process on the degradation of genetic material, specifically on blood and saliva samples, compared to the same type of samples deposited on the surface. Saliva and blood samples were methodically positioned on a white cotton T-shirt, with half of the samples being buried and the other half placed on the surface of the soil within the same quadrant of the box. The degradation was analysed using two complementary approaches: genetic material quantification and genetic profile analysis of autosomal markers, considering also physicochemical factors such as humidity, temperature, and pH. Preliminary results indicate a significant interaction between time, sample location, and sample type in driving genetic loss. Notably, a significant, irreversible loss of genetic material was observed within an average of five weeks for both sample types when buried. Despite the preliminary nature of these findings due to the absence of experimental replicates, they provide a critical forensic baseline for estimating the viable window for DNA profiling in buried evidence.

**Supplementary Information:**

The online version contains supplementary material available at 10.1007/s00414-026-03775-4.

## Introduction

The burial of corpses, or evidence, has long represented both a significant challenge and a valuable investigative lead within forensic science. Criminal cases, like the “Buried Body cases” (USA, 1973), the case of the buried bodies in Fortaleza (Brazil, 2001), or the case of J. Canal (Spain, 2003) demonstrate that burial is a recurrent and deliberate strategy for the concealment of victims. While these cases highlight how soil complicates the initial search and subsequent identification, they also show that the burial environment is frequently pivotal in establishing the facts of a case. However, given that burial is one of the most prevalent methods used to hide evidence, it is essential to determine to what extent forensic analysis is affected by the subsoil environment. Specifically, does burial impact biological samples differently than if they were left on the soil surface? In the context of forensic genetics, the question arises as to whether genetic identification will be more, less, or equally compromised by the act of burial. During the identification process, several phenomena—most notably the loss of genetic information through degradation and the biochemical inhibition of analytical techniques—have the potential to obstruct or entirely prevent a successful profile recovery. Within the scope of inhibitors, several substances intrinsic to the soil have been identified as primary impediments to genetic analysis, necessitating a deeper understanding of their interaction with different biological samples.

### Inhibition and genetic allelic loss

Allelic loss results in irreversible action on the genetic material. Due to various factors, certain loci may undergo a process of allele loss, resulting in an incomplete genetic profile. These factors can be endogenous (e.g., postmortem enzyme and chemical damage by endonucleases and free radicals within the cell, as well as hydrolysis in the presence of water) and exogenous (environmental/soil conditions, including excessive heat, humidity, sunlight, pH, and microbial content) [1]. Most of these factors lead to the degradation of genetic material. On the other hand, if what exists is an inhibition of genetic material, it may be possible to recover this information, albeit partially, especially if a possible origin and factors for this inhibition are detected.

There are several types of inhibition, including: binding of the inhibitor to the DNA template molecule; inhibition of the action of the polymerase enzyme; binding to PCR cofactors, such as magnesium, reducing their availability; interference during the PCR reaction, such as denaturation of the genetic material; or even acceleration of the degradation of components of the genetic material amplification reaction [2–5]. This inhibition can be classified as either partial, characterised by a decrease in sensitivity, or total, resulting in false negatives. The impact of this inhibition can be both direct and indirect, affecting the quantification and amplification of DNA, considering “direct”, where the inhibitory factor is directly linked to the genetic material, or “indirect”, where it affects the mechanism that allows us to obtain a result from that genetic material, as in the case of quantification or amplification. Accordingly, three categories of inhibitors can be distinguished: firstly, endogenous inhibitors derived from the tissue being treated (e.g., blood haemoglobin [2, 6, 7]); secondly, environmental exogenous inhibitors originating from the physical environment from which the samples are retrieved (e.g., humic substances—humic/fulvic acids) [8]; and thirdly, exogenous components introduced into the laboratory during sample processing and/or DNA extraction (e.g., EDTA, SDS) [1]. Different analysis methods are performed in the laboratory to try to analyse highly degraded genetic profiles, such as mini-STR analysis, analysis of various SNP panels using new sequencing techniques, or in other cases, when nuclear material analysis is not possible, the study of mitochondrial DNA, which, although it does not allow identification of the individual, allows the exclusion or inclusion of a particular genetic lineage, via the maternal line [1, 6, 9, 10].

### Soil characteristics

To understand the possible degradation of genetic material in biological samples buried in soil, it is important to know the characteristics of both natural soil and garden soil, the one most people come into contact with. The main difference between a natural and a garden soil lies in its degree of modification and optimisation for specific purposes, usually plant cultivation.

Natural soil is what we find in nature, without significant human intervention in its composition. It is the result of thousands of years of formation processes (pedogenesis), influenced by various factors such as the parent rock, the climate in which it is found, the topography and the presence of organisms [11–13]. Concerning the general characteristics of a soil considered to be natural land, it can vary in terms of texture, pH, fertility and colour, as this will depend on the geographical region in which it was first formed [11]. In terms of texture, it can be sandy, clayey, silty or a combination of several, often with different layers (horizons). One of the important factors is organic matter, as it can vary from very rich to arid soils that are very poor in nutrients. Another factor to take into account is the level of compaction, since it will allow air to circulate or not, and the pH values of each soil are fundamental for the development of certain fauna and flora [11, 12, 14].

Typical garden soil, on the other hand, is usually modified and optimised for plant growth. It is usually characterised by an optimised concentration of organic matter, enriched with humus and other components such as pine bark, peat, among others, to retain both nutrients and water [13]. In terms of texture, it is usually a loamy or sandy loam mixture, with a balanced composition of sand, silt and clay, allowing for adequate drainage and aeration of the soil for proper root growth. The pH is usually between 6.0–7.0 [13, 15, 16]. Finally, it should also be noted that many soils used for gardening are treated [17–20] beforehand to reduce the presence of plant pathogens and weed seeds. These treatments can include disinfection and sterilisation.

The genetic analysis of biological samples in contact with or buried in the soil has been the subject of several studies. For instance, Khorwal et al. (2024) [21] studied the environmental factors that can affect the concentration of DNA in blood and saliva stains. During the degradation process, high temperatures are involved, which accelerate a range of chemical reactions, including hydrolysis, oxidation, and enzyme denaturation. High humidity levels can also play a role, as they can facilitate microbial activity and hydrolysis. Additionally, exposure to sunlight, particularly UV rays, can induce photodamage. The type of substrate also affects the degradation process. For example, fabrics can adequately absorb and retain fluids, which can influence the rate and extent of degradation [21]. As outlined by Bogas et al. (2009) [22], analogous factors include temperature, humidity, UV light, and microbial activity. Furthermore, they compare degradation in three types of soil (sand, clay and marsh soil), the last being the most destructive [22].

Other researchers focus their studies on more specific factors. This is demonstrated in the study by Sutlovic et al. (2008) [23], who investigated the interaction of humic acids with DNA and their inhibitory role in PCR [23]. This topic was further examined by Kasu and Shiresb (2015) [8], who, citing the high failure rate in obtaining genetic profiles generated from biological evidence mixed in the soil with humic acid, sought an extraction system based on the elimination of inhibitors [8]. On the other hand, Hernández-Sánchez (2015) examines the impact of temperature and duration on the viability of DNA for genetic profiling in blood samples, concluding that shorter exposure times yield superior amplification outcomes [24]. In another study, Dissing, Søndervang and Lund (2010) explored the survival limits of DNA in blood, finding that it can be amplified for months, even in difficult conditions such as temperatures of up to 45 °C [25].

It is inevitable that, given these surfaces contain inhibitory compounds, other researchers have proposed different methods of DNA extraction for such circumstances. These include the extraction method with BSA (Bovine Serum Albumin) as additive reagent [26], where the DNA recovery rate was high in all the samples tested (blood, buccal and epithelial cells) [27]; as well as evaluations of existing DNA extraction methods (for blood-stained soils) [28] or even reviews of limitations and recommendations for effective extraction (alluding to pH, soil, inhibitors, contaminants, etc.) [29].

In essence, the probability of locating biological samples buried in soil is notably high, as has been previously documented. Although some studies are focusing on this topic, there are still important questions that have yet to be answered, such as how long it takes to observe partial and total allelic loss when analysing biological samples.

In this sense, the objective of the present work is related to the determination of progressive and/or total allelic loss in saliva and blood biological samples, in contact with soil. To carry out this main objective, some specific objectives were determined, such as a) to analyse the degradation of genetic material differentiating between buried samples and samples placed on the soil’s surface; b) to observe if there are differences in genetic degradation between blood and saliva samples; c) to verify if there is any difference in the degradation of genetic material according to the sex of the individual.

## Materials and methods

### Experimental design

The design and development of this work were approved by the Ethics Committee of the San Carlos Clinical Hospital (Madrid, Spain), with internal code 24/446-E, also respecting the ethical principles for medical research on human beings of the Declaration of Helsinki.

The experimental process took place for ten consecutive weeks, from January to March.

### Simulating a garden plot

**Fig. 1:**
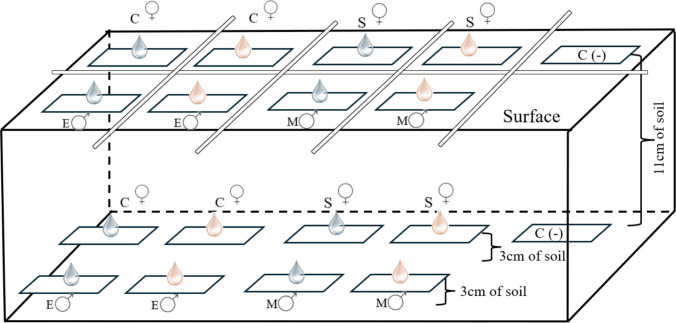
This diagram illustrates the experimental setup used to assess the degradation of biological samples (saliva and blood) on a sample of white cotton fabric from four individuals (two females, C and S, and two males, E and M) for ten consecutive weeks from January to March. The blue drop represents saliva, and the red represents blood

To simulate the effects of burying biological samples in garden soil at a crime scene, a transparent plastic box (30 × 27x18 cm) was selected and filled with soil. The Universal Flower Premium Substrate (10 L) was added in distinct layers. First, a 3 cm deep layer of soil was added to the bottom of the box, and the buried samples were then deposited. Next, another soil layer (11 cm) was added and, finally, a surface sample was deposited (see Supplementary Material 1 and Fig. 1). The garden soil was selected as the study medium due to its prevalence in domestic and urban environments, representing a common scenario for evidence concealment. While soil diversity was restricted by resource and space management for this initial study, this choice establishes a standardised baseline for assessing DNA degradation in a typical, nutrient-rich, and microbially active environment. Future iterations of this project will explore alternative soil compositions, such as sandy or clay soils, to expand the predictive study.

Since the experiment would remain outdoors, seeking to expose it to the most realistic conditions possible, it was necessary to drill holes in the floor of the box to allow water to filter through the rain. The box was placed on a high grid situated over a concrete surface, and the top was covered with two overlapping and crossed grids (water passage), to prevent animal access (Supplementary Material 1).

The surface of the box was divided into equal squares, considering that we had to work with nine extracts per plane (buried/surface) (Fig. 1).

### Support selection

A white cotton T-shirt (80% cotton, 20% polyester) was chosen. Before studying, grids were drawn onto the T-shirt with a pencil to determine where each biological sample should be deposited. Subsequently, the T-shirt was irradiated with 250 nm ultraviolet (UV) radiation to eliminate external genetic material.

### Sample selection

To carry out the experiment, two male and two female individuals were selected. After signing the informed consent form, a blood sample (one drop) was collected from each volunteer by fingerstick with Accu-chek® (Roche) lancets. In addition, a saliva sample (approximately 1 mL) was deposited directly by each subject on the respective support. In total, 18 samples, 9 per plane, were divided into 2 rows, leaving 6 cm of free space between each sample (Fig. 1). These measurements were delineated using a basic structure of small wooden sticks placed on the surface of the box, which simulated each plot (Supplementary Material 1 and Fig. 1). In the same way, each of them was marked out with a "cocktail umbrella" to pinpoint the exact location of each sample and to prevent them from being moved by the wind (Supplementary Material 1). In addition, two controls were also included (the same fabric was used, but without any biological samples), one buried and one on the surface, to assess the presence of any genetic material transference (Fig. 1). Figure 1 illustrates the setting carried out in this work, indicating the individuals and their respective biological samples (saliva and blood) deposited on the cotton fabric, as well as the biological sex of each individual.

### Comparison of physicochemical factors

Every morning, from day 0, around 10:00 a.m. (± 0.5 h), temperature, humidity and pH were measured and recorded, in depth and on the surface, in the centre of the box. Different instruments were used to monitoring the physicochemical variables, such as, a pH and humidity measuring device (*pH-Moisture Meter*, Verdecora) [humidity results of A (dry) – D (wet), and pH of 4 (acid) – 8 (alkaline)], and two thermometers, one for an aquarium compatible with soil (Floating Thermometer PRODAC) (tolerance 0,1 °C), and a terrarium thermometer, together with a hydrometer, which offered temperature and humidity by probes (Sera Reptile Thermometer/Hygrometer) (reduced measurement tolerance 0,1 °C/1% relative air humidity). In addition to monitoring these variables, we monitored daily internal conditions rather than external meteorological data to directly assess the micro-environment acting upon the biological samples, which was moderated by the protective plastic casing.

### Genetic analysis

The extraction of genetic material took place once a week, over ten consecutive weeks, from January to March, in the Department of Legal Medicine, Psychiatry, and Pathology at the Faculty of Medicine of the University of Complutense of Madrid. Before conducting the experimental analysis and burying the tissues with the biological samples, the samples were analysed as would be done in future samplings (considered "week 0"), to have the genetic profiles with which to compare during the experimental work. Subsequently, a weekly schedule was established, with a designated day (every Wednesday) assigned for the collection of a portion of each tissue. Each extraction contemplated 16 extracts and 2 controls – 8 buried and 8 at surface samples, and 1 buried and another at surface control sample. After collecting and cutting the samples, they were left to dry in a controlled environment, inside a biosafety cabinet, at room temperature, until genetic extraction was performed. Sampling was guided by the initial mapping to preserve the limited biological substrate, intentionally omitting presumptive tests (e.g., luminol or amylase detection) to avoid consuming finite material, preventing external contamination, and mitigating potential chemical interference with soil inhibitors.

Genetic material was extracted from saliva samples with the Chelex® (Biorad) method, according to Gomes (2011) [30], and from blood samples with the SPEEDTOOLS TISSUE DNA Extraction kit® (Biotools) protocol, according to the manufacturer's instructions. Genetic material extracts were then stored at −20 °C. Nuclear DNA quantification was performed with Quantifiler™ Human DNA Quantification Kit (Thermo Fisher Scientific™), according to the manufacturer's instructions. It should be noted that this specific kit focuses on total human DNA quantification and does not provide a degradation index (DI) through the comparison of different target sizes.

Genetic profiles were amplified to evaluate possible genetic allelic loss and degradation using the AmpFLSTR Identifiler Plus PCR Amplification Kit (Thermo Fisher Scientific™), with 5 μL of extracted genetic material. Specifically, the tetranucleotide markers D8S1179, D21S11, D7S820, CSF1PO, D3S1358, TH01, D13S317, D16S539, D2S1338, D19S433, vWA, TPOX, D18S51, D5S818 and FGA and the Amelogenin gene were amplified. According to the information in the user manual, this kit allows analytical flexibility using 28- and 29-cycle protocols. Furthermore, due to the size of some of its markers, it allows genetic information to be identified with less than 360 base pairs (bp). Fragments were analysed with an ABI 3730xl Sequencer in the Genomics Unit – CAI Genomics and Proteomics (Faculty of Biological Sciences of the University Complutense of Madrid) and then analysed with GeneMapper™ Software 5 (Thermo Fisher Scientific™).

### Statistical analysis

Statistical analysis of the data was performed using JASP software (version 0.9.5). The main objective was to evaluate the effect of the factors Time, Location, Sample Type, and Gender on the dependent variable, Genetic Information Loss. It wasn't possible to carry out an ANOVA or linear regression analysis with the humidity, pH or temperature values, as there isn't one value per sample; there is one value for the whole box, distinguishing only between buried and surface. There are not enough variance values to carry out this analysis, so these results can only be analysed from a qualitative point of view.

1. Repeated Measures Analysis of Variance (ANOVA)

To test the main effects and interactions between factors, a repeated-measures ANOVA was conducted with the following configuration:

- Dependent Variable: Loss of Genetic Information (considering a complete profile has 32 alleles).
- Intra-subject Factors (Repeated Measures):

Time (10 levels: W1 to W10).

Location (2 levels: Buried and Surface).

Sample Type (2 levels: Blood and Saliva).

- Between-subjects Factor:

Gender (2 levels: Female and Male).

2. Post-hoc Tests

For all significant effects and interactions (with *p* < *0.05*), post-hoc comparison tests were performed. Bonferroni correction was used to adjust the p-values and control the Type I error rate (false positives, i.e., considering that there may be a significant association when in fact there is none), ensuring that the conclusions were robust.

## Results and discussion

### Physicochemical factors

While the primary objective of this research was to evaluate the effect of degradation of genetic material in buried human biological samples (blood/saliva), it was also important to identify several factors that could influence the effect of degradation, and that may alter the obtaining of a genetic profile.

The experimental soil's surface and internal temperatures were recorded, as well as the values of moisture and pH. Table 1 synthesises the obtained values for physicochemical factors.

**Table 1:** Summary of the values measured for pH, humidity and temperature during the ten weeks of the study, measured at 10:00 a.m. (± 0.5 h). Humidity: A (dry) – D (wet)

	**Average value**			**Maximum value**			**Minimum value**		
	**pH**	**Humidity**	**Tº**	**pH**	**Humidity**	**Tº**	**pH**	**Humidity**	**Tº**
“Buried”	6–7	B-C	9.4ºC	6–7	C-D	15ºC	6	B	4.9ºC
“Surface”	7	A	16.4ºC	7	A	26.9ºC	7	A	9.5ºC

The temperature was recorded, both buried and at the surface. Preliminary results indicate that the maximum temperature recorded at the surface (26.9ºC), although relatively high for winter, is not enough to cause denaturation of the DNA chains, which happens at temperatures between 90 and 96ºC, approximately [1, 9]. If temperature has influenced genetic degradation and loss, everything points to a combination of factors and not temperature alone.

After estimating the pH values, it is possible to confirm the stability of this physicochemical parameter, both on the surface (neutral pH = 7) and below ground (burial). The values ranged between 6 and 7 (Table 1). However, likely, the pH value was not the most suitable for the preservation of genetic material, as in the laboratory, pH = 8 is often used. As with temperature, analysing this parameter in isolation from other factors can lead to problems in the conclusions of any similar study.

The humidity record exhibited a comparable trend to that observed in temperature. In this case, the degradation of genetic material was associated with elevated values. Furthermore, this physicochemical parameter promotes the proliferation of microorganisms, which can impact degradation over time [19].

With regard to soil moisture, Table 1 describes the results obtained for buried samples, between B-D, and an A result for the surface. A thorough analysis of the experimental procedure reveals that the surface was dry, and the inner zone was wetter. However, a comprehensive analysis of both physicochemical parameters (temperature and humidity) indicates that temperature significantly impacts the dryness or wetness of each experimental zone.

The soil type was selected to simulate one of the most common open scenarios in forensic investigation, with a methodology where the described physicochemical parameters (temperature, humidity and pH) are controlled. The utilisation of this specific soil type was important in conducting this experiment. The objective was to ascertain whether genetic profiles are susceptible to being lost due to the presence of inhibitors within the soil.

Upon analysing the physicochemical factors in question, no parameter can be identified as extreme or as clearly causing degradation and loss of genetic information. It is more likely that all three factors lead to the development of microorganisms [29] whose activity causes progressive genetic degradation. Conversely, the presence of fertiliser in the garden soil, together with its other components, should have greatly affected the acquisition of genetic information through inhibitory mechanisms. It would be highly recommended to repeat the same study using soil considered to be 'poor' in nutrients, for example.

### Genetic material quantification

Information on the quantification values obtained during this study can be found in the supplementary material. The slope values (−3,3) indicate a perfect quantification reaction efficiency, indicating that the values obtained are reliable [1]. Only the weeks in which quantification values were obtained are indicated. Table 2 summarises the quantification values obtained in this study.

**Table 2:** Summary table showing the only quantifications obtained for nuclear DNA (ng/µl). SG: blood; SAL: saliva; *E*: buried; *S*: surface; _1_, _2,3_ and _4_: weeks

“Buried Blood”	Nuclear DNA (ng/µl)	“Surface Blood”	Nuclear DNA (ng/µl)	“Buried Saliva”	Nuclear DNA (ng/µl)	“Surface Saliva”	Nuclear DNA (ng/µl)
M_1_ SG *E*	0.0068	M_1_ SG *S*	0.0686	M_1_ SAL *E*	0.1059	M_3_ SAL *S*	0.0039
		M_2_ SG *S*	0.0027	M_2_ SAL *E*	0.0359	M_4_ SAL *S*	0.0005
		S_2_ SG *S*	0.0007	C_1_ SAL *E*	0.0616	C_1_ SAL *S*	0.0056
		C_3_ SG *S*	0.0001				
		E_2_ SG *S*	0.0003				
		E_3_ SG *S*	0.0072				

The majority of results with detectable quantification are from surface blood (six quantifications), followed by buried saliva and surface saliva (three quantifications each); only one quantification is observed for buried blood. These results are in line with those obtained with the genetic results, where the best results are seen for the genetic profiles obtained from blood on the surface (Supplementary Material 3). The highest value is found in week 1 for M1SGS (0.0686 ng/µl), and the lowest value is found in week 3, also for surface blood (C3SGS) (0.0001 ng/µl).

As can be seen, the quantification values are unavailable for most of the samples, and the IPC (Internal PCR Control) values were ‘indeterminate’. Given the experimental design, this lack of values could be associated with the presence of soil inhibitors [8], affecting both buried and surface samples in contact with the soil. In the context of this work, we were aware of the possibility of not obtaining results, possibly due to the inhibition of the genetic material; however, it was a surprise to detect this effect so severely in the first week. No results were obtained for the quantification of genetic material after the fourth week of extractions.

It is important to note that, given that this work aimed to verify how the burial or surface exposure of biological samples affected the degradation of genetic material, neither the extraction nor the amplification analysis protocols were modified. This was in order to avoid the introduction of further variables and to ensure that the effect of the soil on the deterioration of genetic material could be observed directly.

### Biological sex

Although the study started from the basis that there were no differences between women and men in terms of stability and degradation of genetic material, a preliminary insight into this subject was obtained. The results (SMTable 1. Allelic loss and S.M. Table 3) indicate that variations in degradation cannot be attributed to biological sex, as something intrinsic, but to other factors, including physicochemical and/o biological factors, such as the possible intervention of microorganisms in the degradation of genetic material in contact with the soil. It is worth noting that during the final week, the presence of diminutive shells of undetermined provenance became evident, as illustrated in Supplementary Material 1. These observations can be attributed to the favourable environmental conditions, including light, temperature, humidity and pH, that prevailed during the extraction process. This was an unexpected factor, although in forensic investigations, biological factors are generally present.

### Genetic analysis

#### Genetic loss of information

**Fig. 2:**
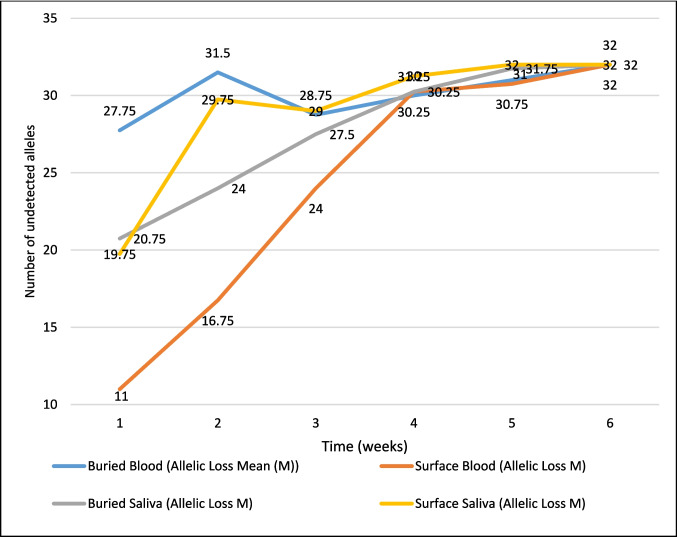
Mean allelic loss variation. The graph depicts the progressive increase in the number of undetected alleles (out of a maximum of 32) over a five-week period for each sample type and environmental condition. Data points represent the mean value for the four studied individuals for Buried Blood (BB), Surface Blood (SB), Buried Saliva (BS), and Surface Saliva (SS), each week. The steeper gradient observed in buried samples highlights the accelerated degradation typically associated with soil-exposure environments

**Fig. 3:**
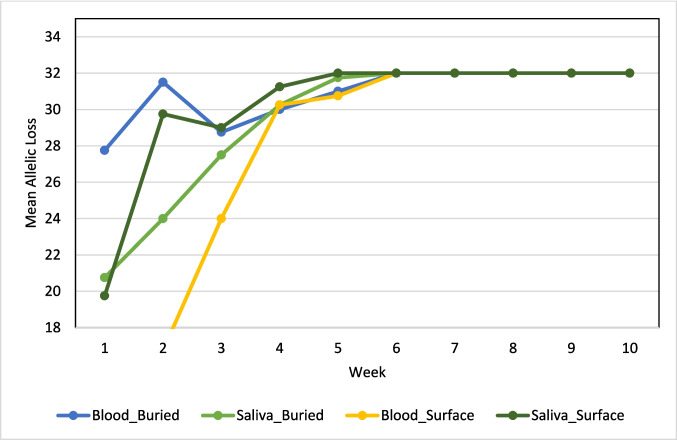
Evolution of mean absolute allelic loss. For visual clarity, error bars are omitted; detailed values are provided in Table 3

Although the quantification results (Table 2 and Fig. 2) indicated severe limitations, the subsequent amplification results enabled the weekly verification of the genetic material degradation (Table 3 and Fig. 3). A possible explanation for the quantification results (‘indeterminate’ from the second week onwards) and the obtaining of genetic results (despite their gradual degradation) could be that the genetic extract is not a homogeneous sample and, in this case, the presence of inhibitors inhibits the quantification of the genetic material but does not prevent the total amplification of the genetic material because it is somehow diluted to carry out the amplification. If the quantification inhibition values in the amplifications had been taken into account, the genetic extract values could have been reduced, and the volume of water increased to dilute the presence of the inhibitors. A lower allelic loss could be observed over the weeks. Without distinguishing between buried and surface samples, allelic loss is more pronounced from week 2 in blood and saliva Fig. 3.

**Table 3:** Mean absolute allelic loss (± SD) over a 10-week period for blood and saliva samples from 4 individuals

Week	Blood Buried		Saliva Buried		Blood Surface		Saliva Surface	
1	27,75 ±	5,19	20,75 ±	9,07	11 ±	14,85	19,75 ±	13,52
2	31,5 ±	0,58	24 ±	14,70	16,75 ±	7,41	29,75 ±	3,86
3	28,75 ±	1,26	27,5 ±	3,70	24 ±	4,32	29 ±	4,08
4	30 ±	1,83	30,25 ±	1,26	30,25 ±	0,50	31,25 ±	0,50
5	31 ±	1,41	31,75 ±	0,50	30,75 ±	1,26	32 ±	0,00
6	32 ±	0,00	32 ±	0,00	32 ±	0,00	32 ±	0,00
7	32 ±	0,00	32 ±	0,00	32 ±	0,00	32 ±	0,00
8	32 ±	0,00	32 ±	0,00	32 ±	0,00	32 ±	0,00
9	32 ±	0,00	32 ±	0,00	32 ±	0,00	32 ±	0,00
10	32 ±	0,00	32 ±	0,00	32 ±	0,00	32 ±	0,00

To visualise this trend, Table 3 summarises the mean absolute allelic loss and the corresponding standard deviation for each condition over the study period.

All samples exhibit a rapid, non-linear increase in allelic loss during the first five weeks, tending towards maximum loss by week 6. Contrary to initial expectations, surface blood samples exhibited the highest resilience, maintaining lower allelic loss values during the first two weeks before a sharp increase. Conversely, buried blood samples showed the most rapid degradation, initiating significant allelic loss immediately from week 1. Saliva samples, regardless of burial or surface exposure, displayed a steep rate of degradation. This visual trend confirms that environmental factors and sample type significantly influence the rate of DNA degradation, with maximum data loss occurring rapidly within the study timeframe.

In this study, the degradation of the genetic material was functionally assessed through the success rate of STR profiling. Due to the advanced state of degradation, particularly in buried samples, extensive allelic drop-out was observed. This widespread loss of genetic information precluded a statistical comparison between small and large molecular weight alleles, common in standard degradation index analyses. Therefore, the inability to generate a full profile was considered a direct pragmatic indicator of irreversible macromolecular damage caused by the environmental conditions.

As a procedural distinction, it should be mentioned that, when making cuts during the extraction, the blood was visible and distinguishable, while the saliva, although it was delimited in an initial circle, was not easily recognisable. This could answer why there is a greater allelic loss in saliva than in blood (SM Table 1. Allelic loss). This procedural difficulty, combined with the lack of a protective matrix like the cellular structure of blood, explains why saliva displays a steeper degradation curve from the outset. In contrast, the visual identification of blood on the surface allowed for more precise sampling, contributing to its observed resilience in the initial stages of the study.

Although blood samples inherently contain a higher concentration of genetic material compared to saliva, the results demonstrate that environmental exposure plays a decisive role. As shown in Fig. 2, while surface blood maintains greater stability during the first two weeks, buried blood degrades as rapidly as saliva samples, suggesting that the soil environment levels the degradation rate regardless of the initial biological matrix (Table 3, Figs. 2 and 3). When starting with fresh samples, as was the case in this study, it is unlikely that the nature of the sample itself leads to it degrading quickly; rather, it is more likely to be due to surrounding factors, such as physicochemical factors and microorganisms. Two examples of genetic profiles showing considerable allelic losses for C_1_SAL*S* (week 1, saliva, surface) and C_1_SG*E* (week 1, blood, buried) can be found in Figs. 8 and 9 of the Supplementary Material.

It is also worth noting that the extraction controls have always appeared uncontaminated. This indicates that there was no laboratory contamination, but it also indicates that there was no transfer between tissues, either horizontally (between buried samples; and between surface samples) or vertically (between surface and buried samples), so it could be concluded that the soil did not act as a means of transferring nuclear DNA, even though it had rained for a few days and the ground was momentarily flooded.

Observing Table 3, Figs. 2, 3 and SM Table 1, results reveals that substantial allelic loss can be detected as early as the third week, as in some cases, information from only three out of sixteen markers is observed. It is important to note that, from the fourth week onwards, genetic profiles become less evident, and only a limited number of alleles can be discerned. This result is of particular relevance, given that four weeks is a brief period in the context of real-world investigations involving the presence of biological samples in contact with the ground, whether buried or not. It is essential to emphasise that this result cannot be extrapolated to the analysis of cadavers or cadaveric remains, since the genetic concentration is incomparable. However, it may be valuable information to take into account when investigating material evidence that may contain biological samples, such as blood or saliva. A recent study from Thomas et al. (2019) demonstrates the potential for the recovery of genetic material for a period of up to approximately five months if the sample is buried in the soil [31, 32]. However, it is important to note that the genetic material analysed in that study is mtDNA. Consequently, the observed differences in results between the present study and that of Thomas may be attributable to variations in the concentrations of mtDNA and nuclear genetic material [31]. Our findings reinforce this distinction, as nuclear DNA loss was almost total by week 4, particularly in buried samples, contrasting with the longer persistence typically reported for mitochondrial DNA in similar burial scenarios. Similar results were previously obtained by Emmons et al. (2017), who refer to the difficulty in obtaining nuclear DNA and the fact that mtDNA is obtained at different times of analysis [33, 34]. In the event of an investigation within a real forensic scenario, it would be recommended that the extraction protocol be considerably modified, with methods that take into account the evident presence of the inhibitors [24]. The potential application of forensic light in detecting saliva and blood should be considered [34, 35], though the challenge of discerning these substances when mixed with soil is a concern. As previously mentioned, it would be advantageous to reduce the volume of extract [36, 37] to decrease the concentration of inhibitors.

One important observation to consider in future work is the 'resistance' of certain genetic markers in this type of research. In each scenario (buried saliva, saliva on the surface, buried blood and blood on the surface), four markers seem to be more resilient than the others: D8S1179, D3S1358, D19S433 and the amelogenin marker (Supplementary Material 3). This observation is significant as it suggests that even in the presence of inhibitors, these genetic markers may still provide crucial information during the first three weeks. However, as shown in Figs. 2 and 3, the rapid degradation observed thereafter suggests that even these markers will likely be lost by the fourth or fifth week in buried samples" This behaviour would be expected concerning the autosomal markers, as they are the 'smallest' markers in each DYE and would therefore be the most likely to provide information in cases of genetic degradation and inhibition.

### Statistical analysis

In this section, statistically significant results will be analysed. The full results can be found in the supplementary material.

1. Temporal Progression (Time: p < 0.001)

Time: F (9,18) = 12.554, p < 0.001, (< p = 0.05) (S.M. Table 2), suggesting there is a statistically significant difference in genetic information loss over time. Genetic loss changes significantly over the 10 weeks.

The highly significant effect of time on allelic loss (*F* (9,18) = 12.554, p < 0.001) confirms that DNA degradation follows a measurable chronological progression rather than an erratic pattern. From a forensic perspective, this predictability is crucial for estimating the 'viability window' of biological evidence. It provides a reliable basis for managing expectations in cold cases or delayed exhumations, where the time elapsed since deposition is a primary determinant of profiling success.

2. Statistical Analysis of Degradation Factors

The analysis of interaction effects revealed complex dependencies between environmental conditions and biological matrices• Location * Sample Type: F(1,2) = 18.888, p = 0.049, (< p = 0.05).

This indicates that the differential impact of burial *versus* surface exposure on genetic integrity is not uniform across different biological fluids. Specifically, the resilience of blood compared to saliva varies significantly depending on the depositional environment, suggesting that the intrinsic biochemical structure of the matrix could dictate its interaction with external soil-induced stressors.

• Time * Location: F(9,18) = 2.722, p = 0.034, (< p = 0.05). This result may confirm that the rate of DNA degradation is not constant but rather accelerated by the burial context over the 10-week period. This finding is crucial for forensic estimations of deposition time, as it demonstrates that soil acts as a catalyst for hydrolytic and enzymatic cleavage, significantly reducing the viability window of buried evidence compared to surface-exposed samples.

• Location * Sample Type * Time: F(9,18) = 3.208, p = 0.017, (< p = 0.05).

This result highlights that the pattern of allelic loss is uniquely determined by the synergistic effect of all three factors. The degradation profile of buried blood differs fundamentally from that of buried saliva, as well as from its surface counterparts. This statistically demonstrates the necessity for multifactorial models in forensic genetics, as the survival of DNA profiles relies on the unique dynamic interaction between the specific biological matrix, the depositional context, and the time elapsed.

3. Post-Hoc Analysis of Degradation Thresholds

To understand the exact moments of significant genetic loss, post-hoc comparisons were conducted. The comparison between surface samples at Week 4 and samples from both locations at Weeks 6–10 proved highly significant (*p* < *0.001*) (S.M. Table 7). This suggests that Week 4 represents a critical threshold, after which the possible protective mechanisms of surface exposure, such as drying, are overwhelmed, leading to rapid degradation comparable to that of buried samples.

Further analysis of the interactions between Biological Sex* Location*Sample type, and time confirmed that for all matrices—buried blood, buried saliva, surface blood, and surface saliva—the degradation between Week 2 and the final weeks (6–10) is highly significant (*p* < *0.001*) (S.M. Table 9). This confirms that the first fortnight post-deposition is the period of highest forensic yield, regardless of the environment.

Direct comparisons between the four groups at Week 2 showed significant differences (*p* < *0.05*), highlighting that the initial rate of degradation is matrix- and environment-dependent. Notably, the comparison between Buried Blood at Week 2 and Surface Saliva at Week 5 was highly significant (*p* < *0.001*) (S.M. Table 10), reinforcing that location impacts DNA viability more rapidly than time alone.

Finally, the comparison between baseline measurements and the stabilisation phase (Week 2 vs. Week 5, *p* < *0.001*) confirms that by Week 5, the samples reach a state of irreversible genetic loss. This statistical stabilisation marks the point at which hydrolytic and enzymatic cleavage has progressed to a degree where generating informative profiles is technically impossible, establishing a clear scientific rationale for the limits of profiling success in forensic casework.

4. Analysis of Near-Significant Tendencies

A near-significant trend was observed when comparing surface blood to surface saliva (*p* = *0.051*). Although slightly above the conventional 0.05 threshold, this value is highly relevant as it suggests that, in the absence of aggressive soil-induced stress, blood tends to be more resilient than saliva. This borderline difference likely contributed to the significance of the global ANOVA model, reinforcing that the intrinsic biochemical composition of biological matrices—specifically the cellular density and clotting ability of blood—provides superior stability against atmospheric factors compared to the enzymatic content of saliva.

The preliminary findings of this study provide a comprehensive insight into the degradation kinetics of biological evidence, highlighting that DNA survival is not merely a function of time, but a complex synergy between the biological matrix and the depositional environment. The statistical analysis demonstrates that while all samples degrade over time, the “aggressiveness” of the burial context catalyses this process, reducing the effective profiling window to approximately two weeks.

From a forensic standpoint, this study establishes a scientific basis for prioritising certain types of evidence based on location. The demonstrated rapid collapse of buried saliva compared to surface-exposed blood suggests that investigators should focus on salivary traces within the first 72 h in burial scenarios, whereas surface bloodstains offer a wider, though still finite, temporal window for successful analysis.

Furthermore, the stabilisation of degradation by Week 5—resulting in irreversible genetic loss—provides a crucial metric for the judicial system. It scientifically validates the inability to generate profiles from samples found after prolonged exposure, attributing this failure to complete environmental degradation rather than analytical error.

While recognising the limitations of this study, the preliminary results are important for informing the approach of future research. One of the main difficulties is that the reduced number of samples analysed (n = 4) would have to be increased in the future to carry out a more robust statistical investigation. The data collected here provides an initial insight into the problem, but it is necessary to increase this sample size to extrapolate the findings securely. Another obvious limitation is that, while this study attempts to simulate a real-world scenario, it lacks the capacity to account for all possible variables involved in the degradation of biological samples, both when buried and on the surface. For example, the action of roots or faunal activity can not only cause degradation but also alter the position of the samples. On the other hand, the burial depth in this project does not correspond to that found in all real-world crime scenes. However, the results obtained in this preliminary study are considered relevant as they demonstrate a possible trend to be considered in future research, as well as in real investigations. As a prospect, it would be important to vary not only the type of biological samples and the type of fabric (such as cotton, polyester, silk, or linen) but also the type of soil, now including sand, or more calcareous or clay soils. Despite the high cost, it would also be interesting to investigate the behaviour of degradation and allelic loss daily, which would provide clearer information to help determine when genetic information begins to be lost. Also, it would be highly informative to perform the analysis with an amplification kit that contains inhibitor “flags” or sensors to further refine the biochemical understanding of the degradation process.

Finally, a pertinent question arises regarding the applicability of these preliminary findings to more complex biological matrices, such as cadaveric remains. While this preliminary study utilised isolated biological stains, a whole cadaver introduces endogenous degradation pathways, including autolysis and internal putrefaction, which were not present in our experimental model. However, the “aggressive” degradation kinetics observed in the buried samples underscore the significant role of exogenous factors—specifically soil moisture and microbiota—which would similarly impact a decomposing body. While our results should be viewed as a controlled baseline, they serve as a relevant proxy for understanding how the burial environment interacts with the “*taphonomic island*” of a decaying corpse, suggesting that soil-mediated degradation remains a primary driver of genetic loss even in the presence of internal putrefactive processes.

## Conclusions

Despite the substantial limitations of this study, these preliminary results provide critical insights into the viability of biological evidence. The most significant finding is that DNA degradation in buried environments is not merely a function of time, but a result of the synergistic interaction between the biological matrix and the soil ecosystem, leading to a much faster loss of genetic information compared to surface-exposed samples.

The statistical analysis conclusively shows that factors such as time, sample type, and location are the primary drivers of allelic loss, whereas biological sex does not influence the degradation rate. Furthermore, the preliminary results suggest that no single physicochemical factor was extreme enough to cause degradation in isolation. Instead, it is highly likely that the stable environment within the soil promoted the proliferation of microorganisms, whose metabolic activity led to the destruction of the genetic material.

Another crucial factor was the composition of the garden soil, rich in humic and fulvic acids, which acted as a significant driver for the inhibition of genetic amplification. Finally, the observed lack of genetic material transfer between samples indicates that garden soil may act as a barrier rather than a 'conductor' for DNA “migration”. In conclusion, while these findings are preliminary, they establish a clear scientific rationale for the limits of profiling success, emphasising that the forensic window for buried evidence is significantly shorter and more complex than previously estimated.

## Supplementary Information

Below is the link to the electronic supplementary material.

Supplementary file1 (PDF 1017 KB)

Supplementary file2 (XLSX 364 KB)

## Data Availability

All the data relevant to this research can be found in the supplementary material.
