# Supplementary material for: Unearthing forensic genetics: preliminary results on the ability to generate DNA profiles from buried human biological samples
Source: Int J Legal Med. 2026 Mar 25;140(4):1997–2009. doi: 10.1007/s00414-026-03775-4 (PMC13275538; doi:10.1007/s00414-026-03775-4)

Supplementary Material 1

International Journal of Legal Medicine

Unearthing Forensic Genetics: preliminary results on the ability to generate DNA profiles from buried human biological samples

Susana Gracia-de-Lucas[1], César López-Matayoshi[2,3], Manuel Lozano-García[2] and Cláudia Gomes[2, *]

*Corresponding authors: Cláudia Gomes; clopes01@ucm.es; César López Matayoshi, clopezma@ucm.es

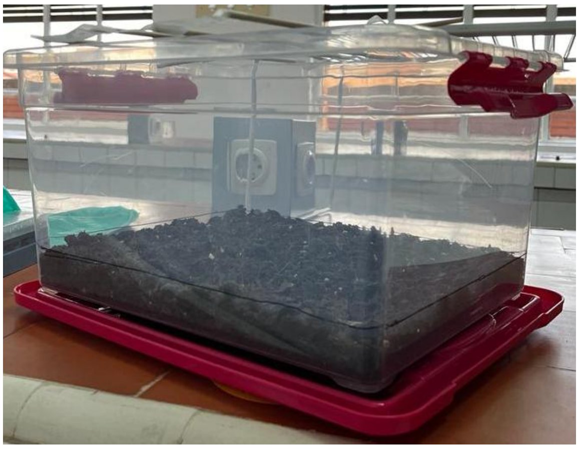

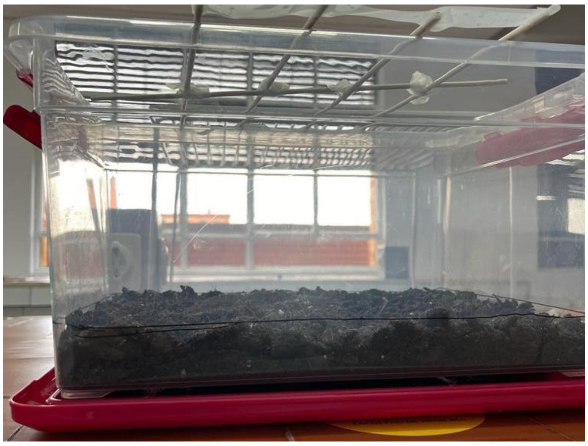

Figure 1 and 2. *Delimitation 3cm^3 of soil from the floor of the box.*

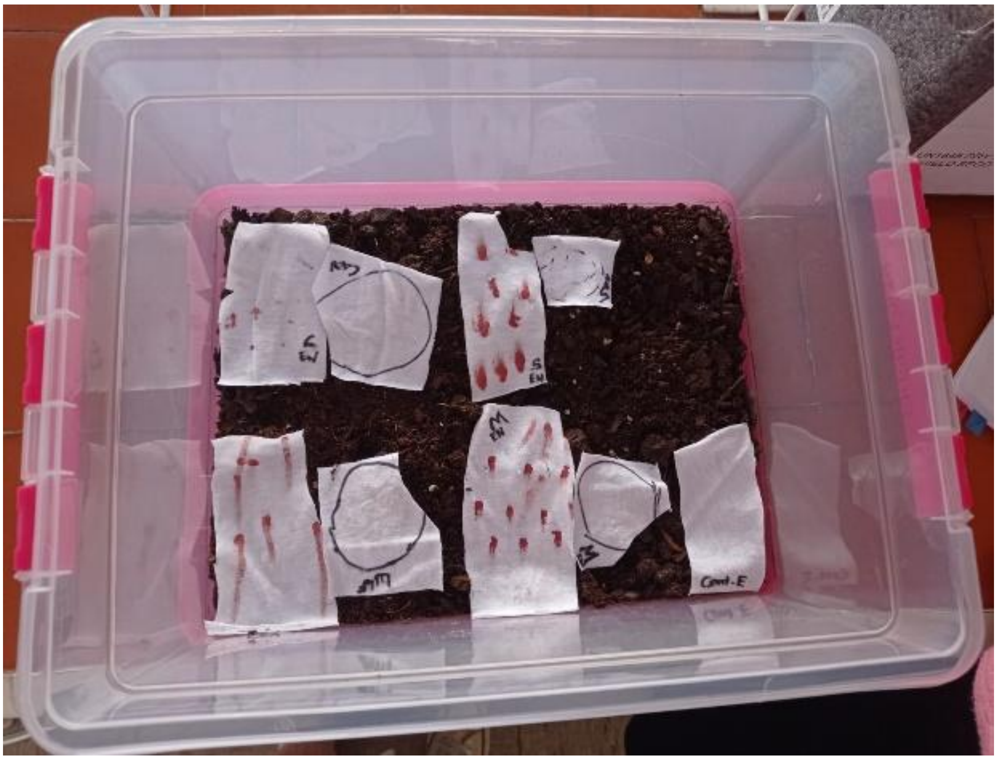

Figure 3. Samples before burial with soil.

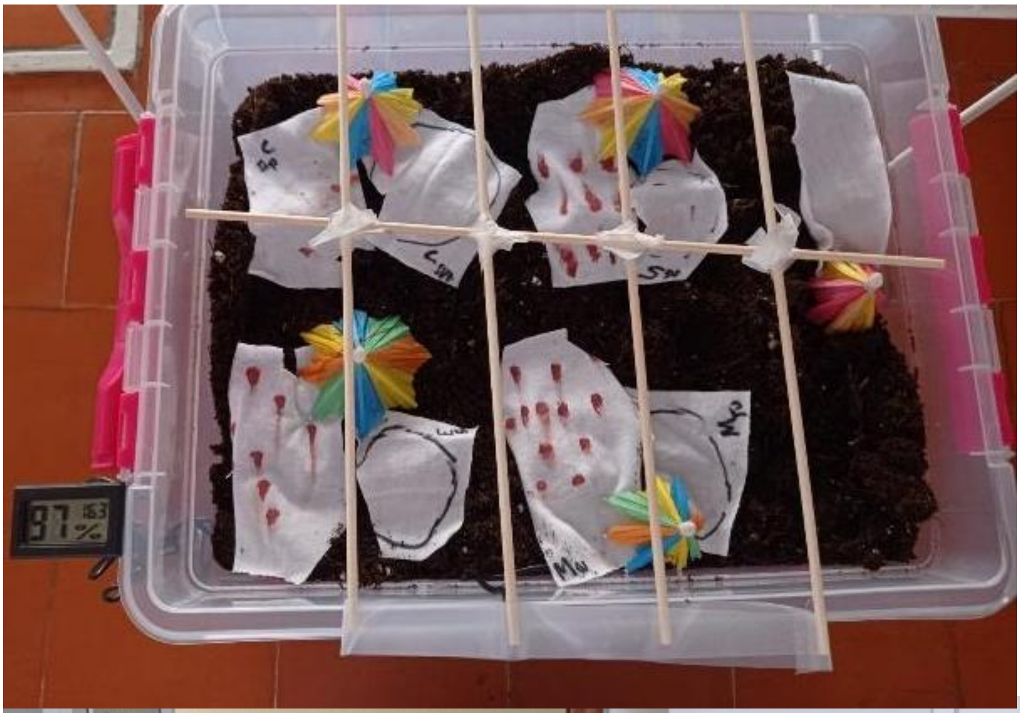

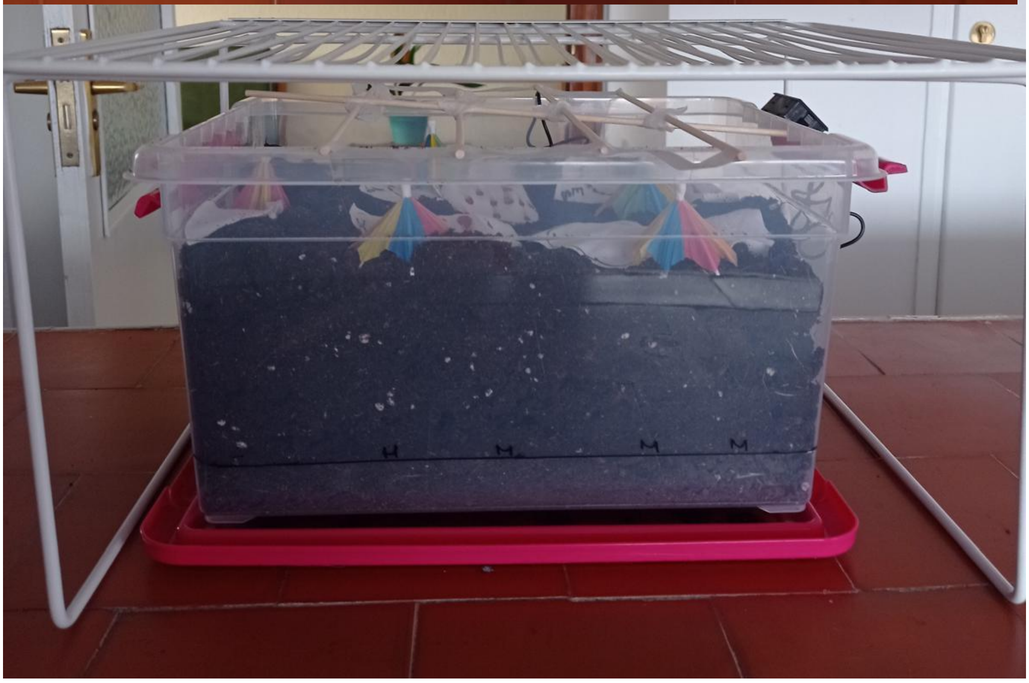

Figure 4 and 5. Experiment with cross-linked grids, to prevent animal interference.

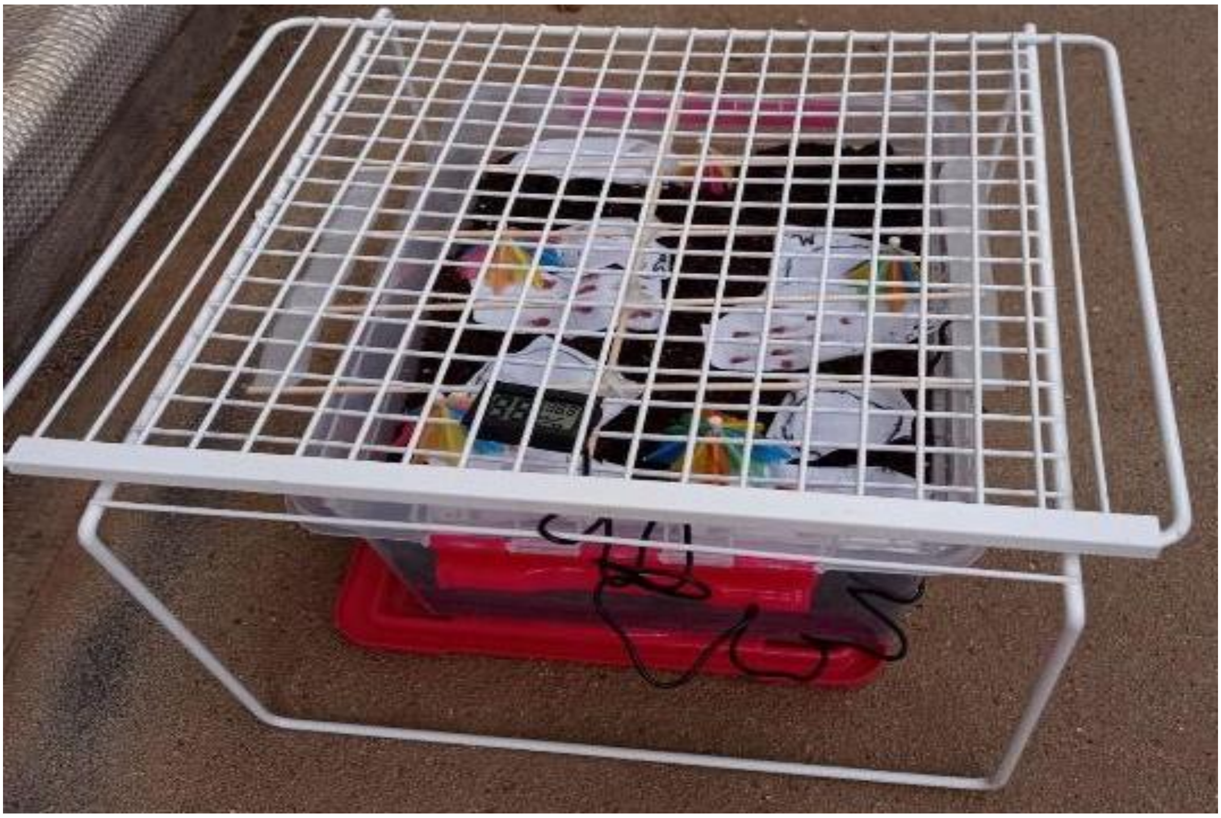

Figure 6. Experiment in its final location outside.

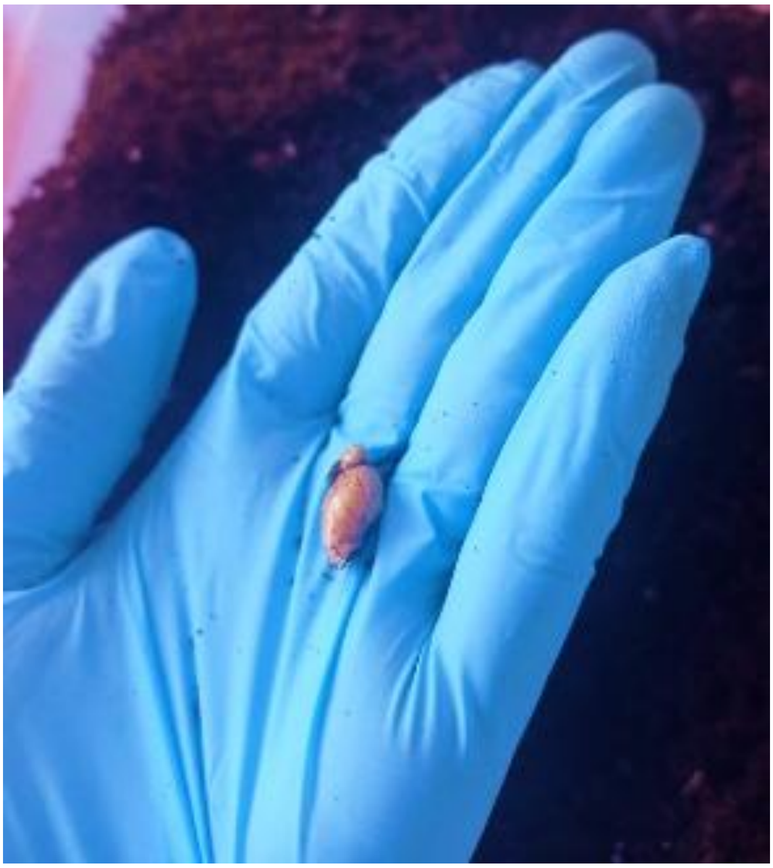

Figure 7: Shell found during the experimental process.

Figure 8. Example genetic profile for C_1SAL*S*, where there is a loss of 4 alleles.

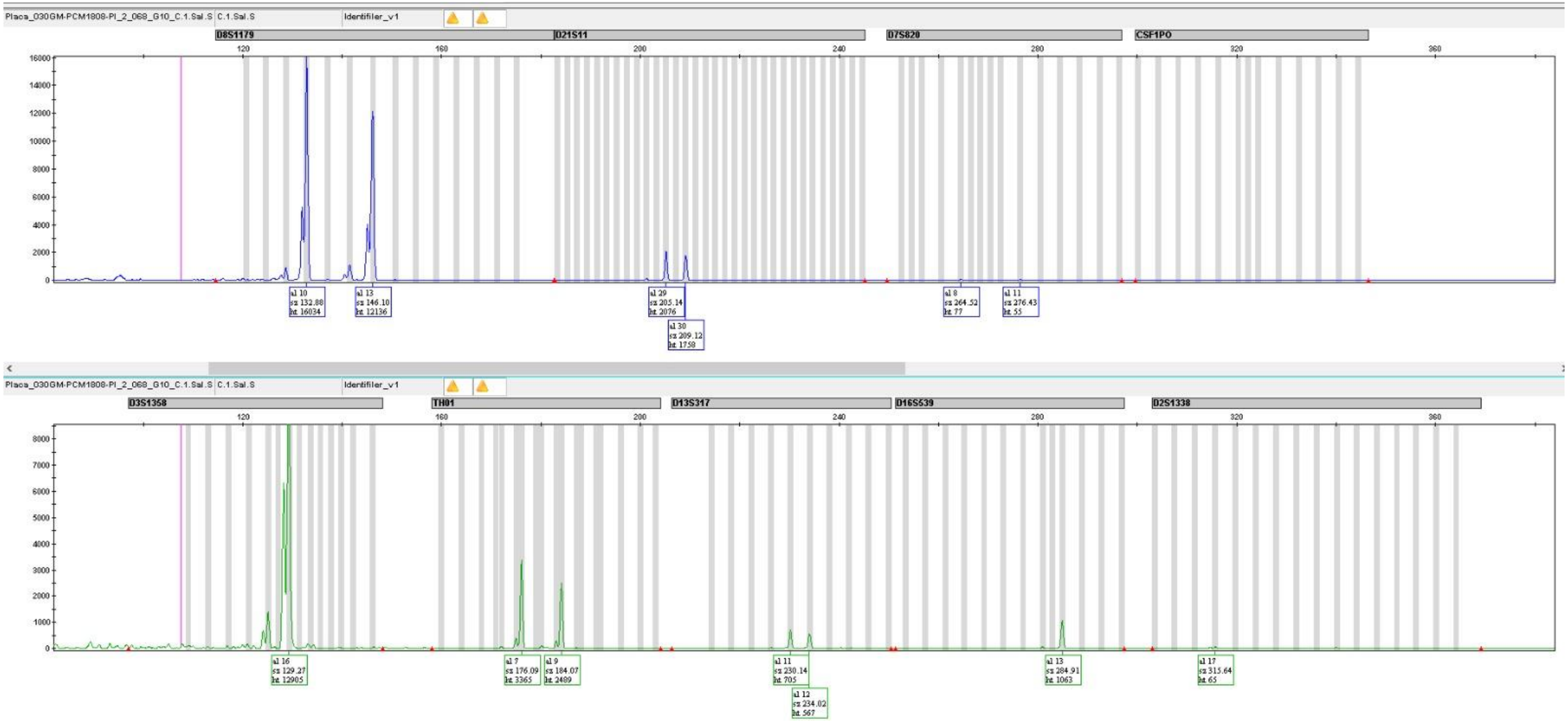

Figure 8. Example genetic profile for C_1SAL<u>*S*</u>, where there is a loss of 4 alleles (continued).

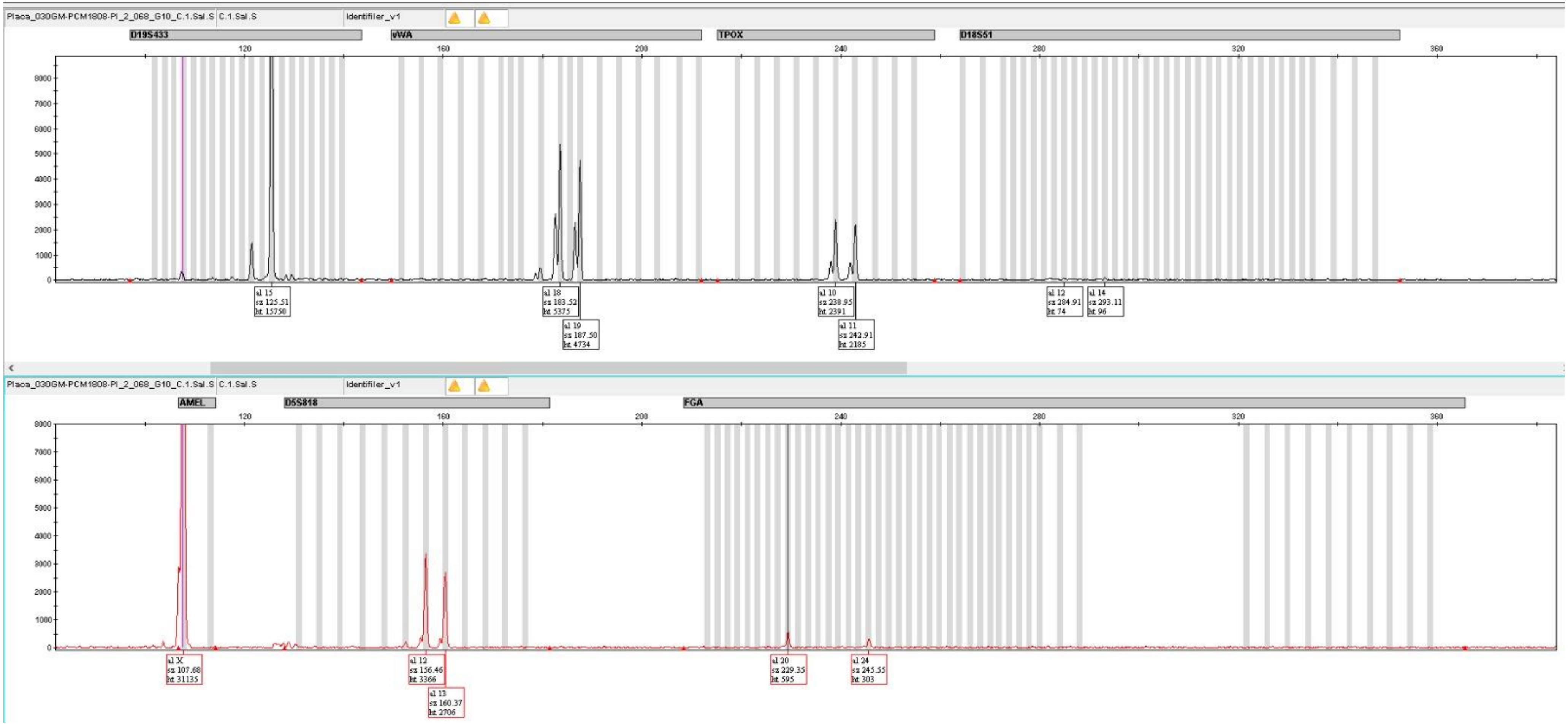

Figure 9. Example genetic profile for C_1SG<u>*E*</u>, where there is a loss of 30 alleles.

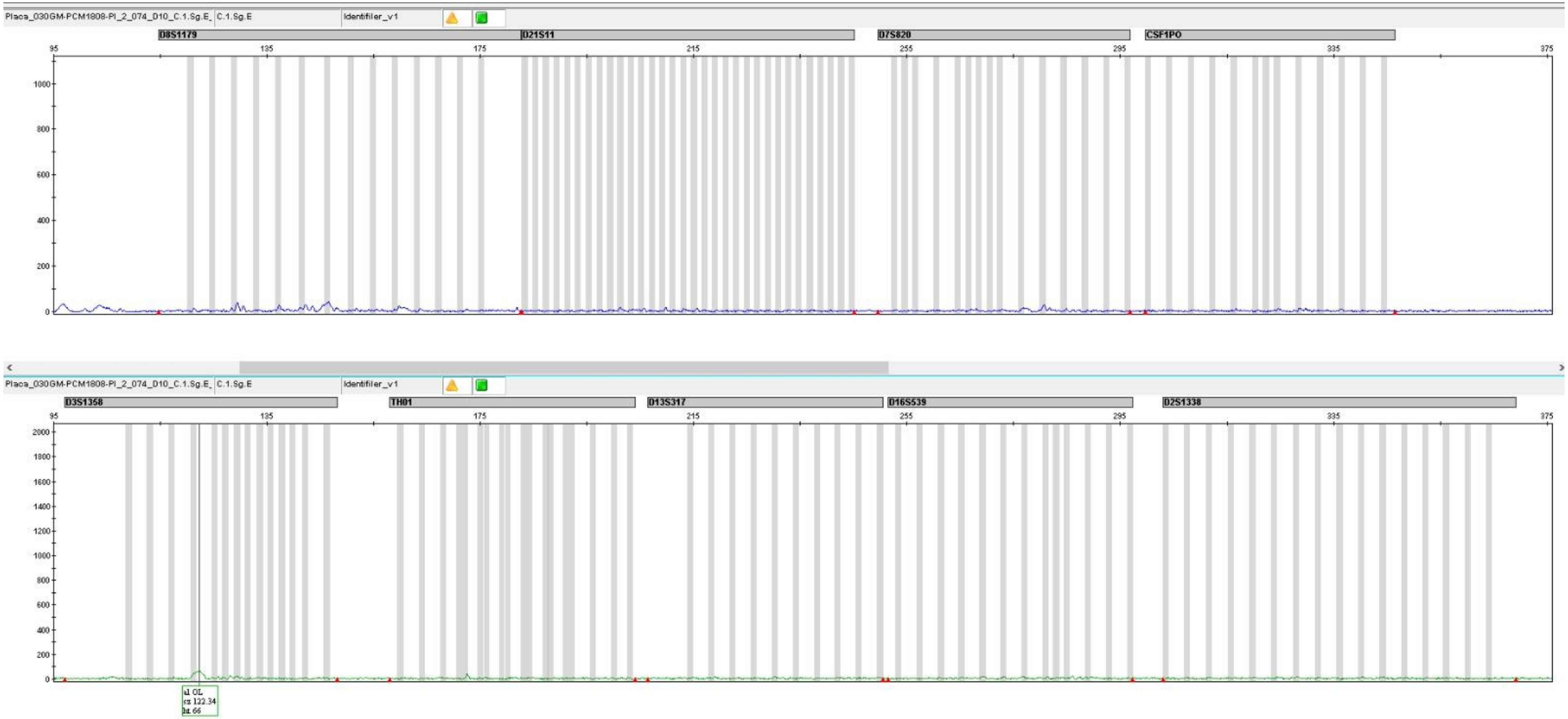

Figure 9. Example genetic profile for C_1SG<u>*E*</u>, where there is a loss of 30 alleles (continued)

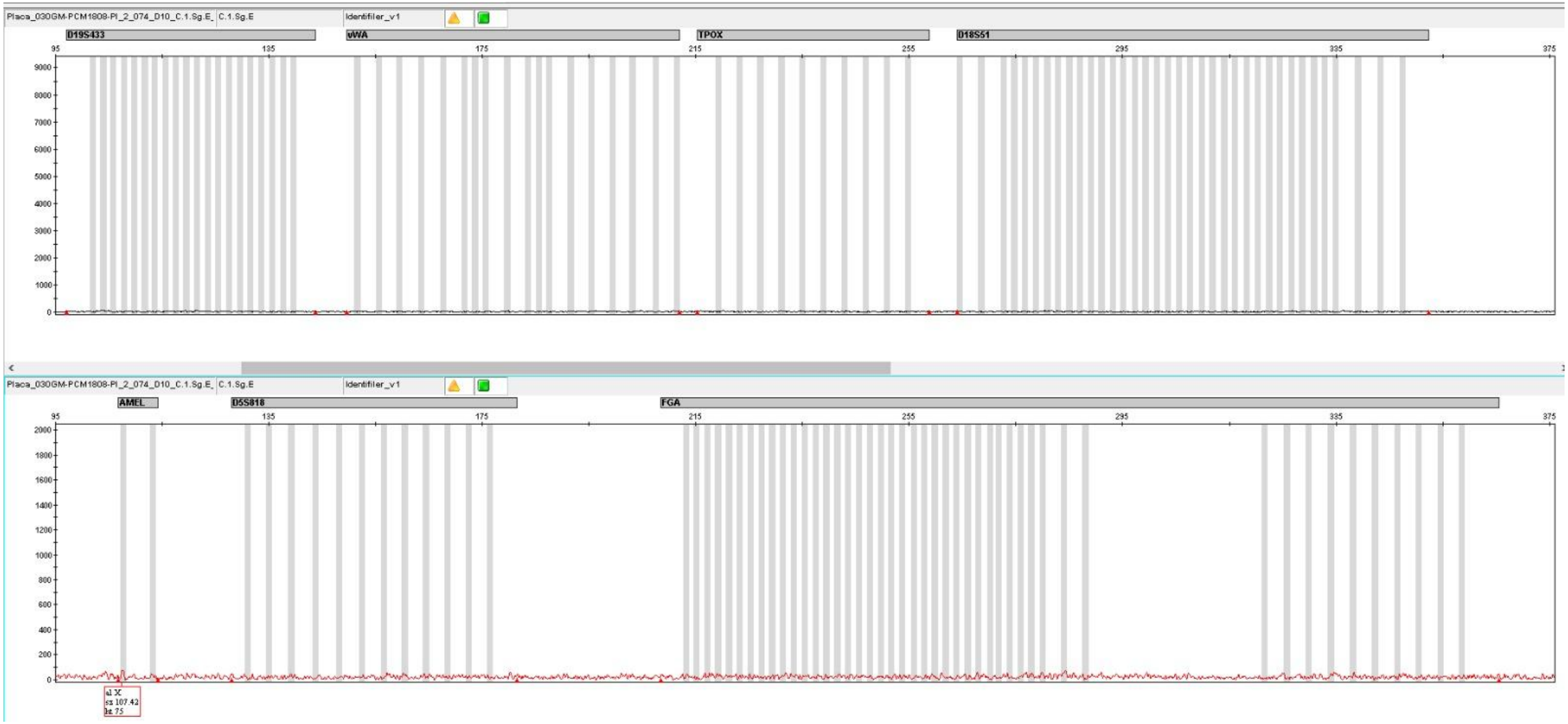

Supplement: Supplementary file 1 — Supplementary file1 (PDF 1017 KB) [file 414_2026_3775_MOESM1_ESM.pdf]
